# Missing WD40 Repeats in ATG16L1 Delays Canonical Autophagy and Inhibits Noncanonical Autophagy

**DOI:** 10.3390/ijms25084493

**Published:** 2024-04-19

**Authors:** Jiuge Tang, Dongmei Fang, Jialing Zhong, Min Li

**Affiliations:** 1 State Key Laboratory of Anti-Infective Drug Discovery and Development, School of Pharmaceutical Sciences, Sun Yat-sen University, Guangzhou 510006, China; 2 Guangdong Provincial Key Laboratory of Chiral Molecule and Drug Discovery, Guangzhou 510006, China

**Keywords:** ATG16L1, canonical autophagy, noncanonical autophagy, WDR domain

## Abstract

Canonical autophagy is an evolutionarily conserved process that forms double-membrane structures and mediates the degradation of long-lived proteins (LLPs). Noncanonical autophagy (NCA) is an important alternative pathway involving the formation of microtubule-associated protein 1 light chain 3 (LC3)-positive structures that are independent of partial core autophagy proteins. NCA has been defined by the conjugation of ATG8s to single membranes (CASM). During canonical autophagy and NCA/CASM, LC3 undergoes a lipidation modification, and ATG16L1 is a crucial protein in this process. Previous studies have reported that the WDR domain of ATG16L1 is not necessary for canonical autophagy. However, our study found that WDR domain deficiency significantly impaired LLP degradation in basal conditions and slowed down LC3-II accumulation in canonical autophagy. We further demonstrated that the observed effect was due to a reduced interaction between ATG16L1 and FIP200/WIPI2, without affecting lysosome function or fusion. Furthermore, we also found that the WDR domain of ATG16L1 is crucial for chemical-induced NCA/CASM. The results showed that removing the WDR domain or introducing the K490A mutation in ATG16L1 significantly inhibited the NCA/CASM, which interrupted the V-ATPase-ATG16L1 axis. In conclusion, this study highlights the significance of the WDR domain of ATG16L1 for both canonical autophagy and NCA functions, improving our understanding of its role in autophagy.

## 1. Introduction

Macroautophagy, also called canonical autophagy, is an important lysosomal degradation pathway that maintains cell and tissue homeostasis by removing damaged and toxic cellular components, including protein aggregates, damaged organelles, and long-lived proteins (LLPs) [1]. Canonical autophagy is associated with the development of numerous diseases, including neurodegenerative diseases, aging, cancer, etc. [2]. Canonical autophagy involves the sequestration of damaged organelles and LLPs by microtubule-associated protein 1 light chain 3 (LC3)-positive isolation membranes to form double-membrane autophagosomes. These autophagosomes then fuse with lysosomes for protein degradation. Autophagosome formation requires a series of core autophagy-related proteins, including Unc-51-like autophagy activating kinase 1 (ULK1) complex and autophagy-specific phosphatidylinositol 3-kinase complex mediating membrane nucleation and phagophore formation, the ATG12~ATG5-ATG16L1 and LC3-phosphatidylethanolamine ubiquitin-like conjugation systems mediating phagophore expansion [3].

The mammalian ATG8 protein family, which includes microtubule-associated protein 1 light chain 3 (LC3A/B/C) and γ-aminobutyric acid receptor-associated proteins (GABARAP/L1/L2), plays a crucial role in the autophagy mechanism [4]. During canonical autophagy, ATG8s bind to the lipid phosphatidylethanolamine (PE) on autophagic precursors [5]. This unique modification is crucial for the selection of autophagic cargo, membrane extension, and closure. Recent studies have shown that LC3 can target not only double-membrane autophagosomes but also other monolayer membrane vesicles of the endolysosome system [6]. These phenomena are collectively referred to as noncanonical autophagy (NCA), as they share a common feature—the conjugation of ATG8s to single membranes (CASM). Numerous studies have demonstrated the significant physiological functions of NCA/CASM, particularly in the immune system, cancer, neurodegenerative diseases, and vision [7,8,9,10]. NCA can be classified into four main types: heterophagy-related NCA which includes LC3-associated phagocytosis (LAP) [11,12], LC3-associated macropinocytosis (LAM) [13,14], LC3-associated endocytosis (LANDO) [15,16], and entosis [17,18]; pharmacological treatment-induced NCA, such as lysosomotropic drugs [19,20,21], Ionophores [22], TRPML1 agonists [23] and drugs driving Golgi ATG8 lipidation [24,25]; pathogenic factor-induced NCA which includes viruses, STING, and bacterial toxins [26,27,28]; other candidate NCA-related processes including microautophagy and LC3-dependent extracellular vesicle loading (LDELS) [29]. 

The lipidation of LC3 is required for both canonical and noncanonical autophagy (NCA), and this process involves ATG12 and LC3 ubiquitin-like conjugation systems. ATG12 is first activated by the E1-like protein ATG7 and then binds to ATG5 via the E2-like protein ATG10 to form the ATG5–ATG12 complex, which is followed by ATG16L1 via its N-terminal ATG5-binding domain to form the ATG12–ATG5–ATG16L1 complex. The final step in LC3 lipidation is performed by the E3-like enzyme ATG12–ATG5–ATG16L1 complex, in which ATG16L1 functions to localize the complex to the autophagy initiation site [30].

In 1993, Tsukada and Ohsumi identified a series of autophagy-related genes (ATGs) involved in the autophagy pathway, including Atg1–Atg15, through a genetic screen in yeast [31]. A subsequent study identified Atg16 (initially referred to as Apg16) in a yeast two-hybrid screen for interactions with Atg12. In 2003, mammalian ATG16L (a homologue of yeast Atg16) was first identified during immunoprecipitation of ATG5 in mouse cells [32]. However, this discovery received little attention until a study found that the absence of ATG16L1 increased susceptibility to Crohn’s disease [33,34]. Since then, many research teams have investigated the role of the ATG16L1 protein in maintaining cellular homeostasis. Atg16 protein in yeast comprises an Atg5-binding domain and a coiled-coil structure [35,36]. In contrast to yeast, the ATG16L protein has an additional C-terminal region comprising seven WD40 repeat sequences that form a β-propeller structure [37]. The mammalian genome encodes two ATG16L proteins: ATG16L1 and ATG16L2. Considering the sequence similarity between ATG16L1 and ATG16L2 is not high and ATG16L2 does not participate in LC3 lipidation during autophagy [38], this study focuses mainly on ATG16L1.

Existing studies have indicated that the WDR domain of ATG16L1 is not necessary for canonical autophagy [27,39,40]. Even in cells with a deficiency in the WDR domain, LC3 lipidation can still occur, and long-lived proteins (LLPs) can still be degraded by amino acid starvation or canonical autophagy inducers such as mTOR inhibitor PP242 [41,42]. p62 is a typical receptor for canonical autophagy, which identifies and recruits proteins to autophagosomes for further degradation in autolysosomes. However, recent studies have shown that there is an inconsistency on this point; the interaction between ATG16L1^ΔWDR^ and p62 is weak, and p62 significantly accumulates in the liver and kidneys of mice with ATG161L1^1−230^, in which the WDR domain is absent [39,41]. Furthermore, WDR domain-deficient mice exhibited Alzheimer’s disease (AD) phenotypes [40], indicating that canonical autophagy might be somewhat blocked when cells were deprived of the WDR domain of ATG16L1. Although autophagic stimulation can induce LC3 lipidation and LLP degradation in cells without a WDR domain, it is unclear whether the WDR domain is necessary for basal canonical autophagy.

Recent studies have shown that the WDR domain of ATG16L1 is essential for supporting NCA/CASM [41,42]. ATG16L1 can be recruited to vesicles containing bacteria and mediate LC3 lipidation through the interaction between the WDR domain and V-ATPase subunits, instead of WD repeat domain phosphoinositide-interacting protein 2 (WIPI2) in xenophagy [43]. Our previous studies have shown that NCA induced by chemicals or drugs such as 2-[5,7-bis(trifluoromethyl)[1,8]naphthyridin-2-yl]-2-(3-chlorophenyl) (AMDE-1), niclosamide, and carbonyl cyanide m-chlorophenyl hydrazone (CCCP) is a ubiquitous phenomenon [24,25,44]. However, the induction of NCA/CASM by AMDE-1 and niclosamide is not related to canonical autophagy signals or lysosome function, despite their ability to suppress lysosomal function and initiate phagophore formation [24,25]. It is currently unclear how these chemicals mediate NCA and whether there is redundancy of WDR for ATG16L1. Therefore, further systematic studies are necessary to identify the regulatory mechanisms of ATG16L1 in mediating canonical autophagy and NCA. This is significant for clarifying intracellular vesicle transport and expanding the ATG16L1 function in mediating different autophagy pathways. 

This study focused on the roles of ATG16L1 and its WDR domain in both canonical autophagy and NCA. The effects of the WDR domain on canonical autophagy were investigated mainly using ATG16L1^ΔWDR^-HeLa cell lines; LC3 lipidation levels and LLP degradation were also tested. We found that the WDR domain is crucial for canonical autophagy as it suppresses basal-level autophagy, stalls autophagic flux, and delays the degradation of p62 when the WDR domain is absent. We also demonstrated that chemical-induced NCA/CASM by AMDE-1 and niclosamide depends on the intact WDR domain of ATG16L1 using a cell model with artificial WDR deficiency. 

## 2. Results

### 2.1. Autophagy Induced by Torin1 and Starvation Dropped Due to the Deletion of WDR of ATG16L1

**Figure 1:**
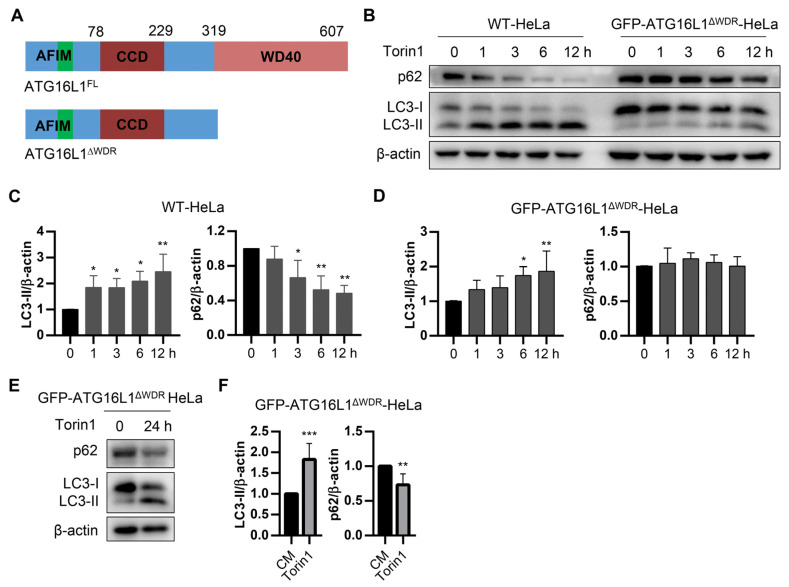
Torin1-induced autophagy in ATG16L1^ΔWDR^ cells is lagged. (**A**) Abridged general view of full length ATG16L1 (ATG16L1^FL^) and ATG16L1^ΔWDR^. AFIM: ATG5 interaction motif, CCD: coiled-coil domain, WDR: WD40 repeats domain. (**B**) WT-HeLa cells and *ATG16L1^−/−^*-HeLa cells stably expressing GFP-ATG16L1^ΔWDR^ were treated with 1 μM of Torin1 for 0, 1, 3, 6, 12 h. p62, LC3, and β-actin were analyzed by immunoblotting. (**C**,**D**) Quantification of LC3-II/β-actin ratios and p62/β-actin ratios in (**B**), *n* = 3. * *p* < 0.05, ** *p* < 0.01. (**E**,**F**) *ATG16L1^−/−^*-HeLa cells stably expressing GFP-ATG16L1^ΔWDR^ were treated with 1 μM of Torin1 for 24 h. p62, LC3, and β-actin were analyzed by immunoblotting and quantified, *n* = 3. ** *p* < 0.01 and *** *p* < 0.001.

Figure 1A shows ATG16L1 with or without the WDR domain. The ATG16L1KO HeLa cells, which stably express GFP-ATG16L1, GFP-ATG16L1^ΔWDR^, and GFP-ATG16L1^1−230^, were designated as GFP-ATG16L1-HeLa, GFP-ATG16L1^ΔWDR^-HeLa cells, and GFP-ATG16L1^1−230^-HeLa cells. WT-HeLa and GFP-ATG16L1^ΔWDR^-HeLa cells were treated with 1 μM of Torin1. The results indicated that the LC3-II accumulation in GFP-ATG16L1^ΔWDR^-HeLa and GFP-ATG16L1^1−230^-HeLa cells was significantly slower than that in WT-HeLa cells (Figure 1B and Figure S1B). Furthermore, the level of LC3-II induced by Torin1 in GFP-ATG16L1^ΔWDR^-HeLa cells was lower compared to that in WT HeLa and GFP-ATG16L1-HeLa cells. In WT HeLa cells, p62 levels decreased significantly after a 3-h treatment with Torin1, while there was no significant reduction in p62 levels after 12 h in WDR-deficient cells (Figure 1B–D and Figure S1A–C). However, p62 was downregulated at 24 h in WDR-deficient cells (Figure 1E,F). 

**Figure 2:**
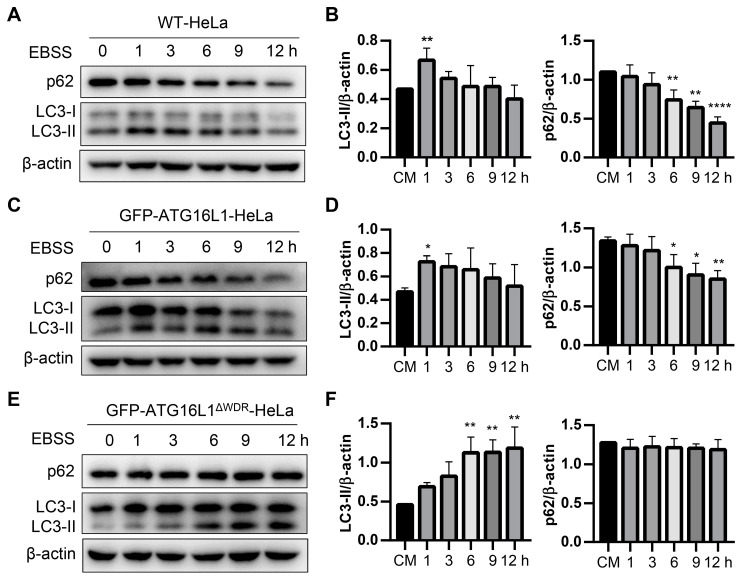
Starvation-induced autophagy in ATG16L1^ΔWDR^ cells is lagged. (**A**–**F**) WT-HeLa cells (**A**,**B**) and *ATG16L1^−/−^*-HeLa cells stably expressing GFP-ATG16L1 (**C**,**D**) or GFP-ATG16L1^ΔWDR^ (**E**,**F**) were incubated with EBSS for 0, 1, 3, 6, 9, and 12 h. Protein levels of p62 and LC3 were analyzed by immunoblotting and quantified, respectively, *n* = 3. * *p* < 0.05, ** *p* < 0.01, and **** *p* < 0.0001.

To evaluate the impact of ATG16L1 overexpression on autophagic activity, we compared WT-Hela cells with ATG16L1-HeLa cells (*ATG16L1^−/−^*-HeLa cells stably expressing GFP-ATG16L1) treated with Earle’s balanced salt solution (EBSS), a commonly used canonical autophagy inducer. The results showed that the levels of LC3-II and p62 levels in both cell types were almost identical (Figure 2A–D), indicating that the ATG16L1-HeLa cells used in this study had properties similar to those of WT-Hela cells. Subsequently, WT-HeLa, GFP-ATG16L1-HeLa, GFP-ATG16L1^ΔWDR^-HeLa, and GFP-ATG16L1^1−230^-HeLa cells were cultured in EBSS for varying durations. The results showed that LC3-II levels significantly increased in WT-HeLa and GFP-ATG16L1-HeLa cells after 1 h of starvation, and decreased after 3 h (Figure 2A–D). However, GFP-ATG16L1^ΔWDR^-HeLa and GFP-ATG16L1^1−230^-HeLa cells showed an increase in LC3-II levels at 6 h, but did not exhibit a decrease even after 12 h of starvation (Figure 2E,F and Figure S1D,E). Furthermore, p62 levels in WT-HeLa and GFP-ATG16L1-Hela cells were continuously degraded after starvation (Figure 2A–D). In contrast, p62 was not significantly decreased in WDR-deficient cells even after 12 h of starvation (Figure 2E,F and Figure S1D,E). 

Taken together, our results suggest that the deletion of the WDR domain of ATG16L1 results in a significant delay in the lipidation of LC3 in canonical autophagy. 

### 2.2. Deletion of the WDR Domain of ATG16L1 Impaired Basal Autophagic Degradation

During canonical autophagy, autophagic cargo is transported to lysosomes for degradation. Previous work has shown that GFP-ATG16L1^ΔWDR^-Hela cells are unable to degrade p62. LLP degradation is a gold standard for measuring autophagic activity. Therefore, we conducted further investigations into the degradation of long-lived proteins. In the synthesis of long-lived proteins, L-azidohomoalanine (AHA) was used intracellularly as a replacement for methionine due to its structural similarity. Then, the amount of residual AHA in the cells was analyzed by flow cytometry after drug administration to reflect the relative degradation of long-lived proteins. 

**Figure 3:**
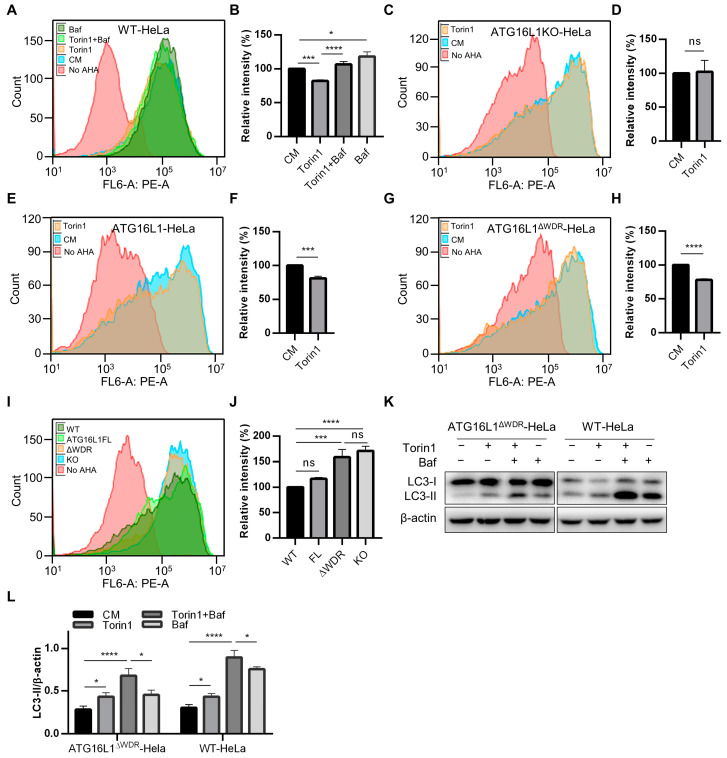
ATG16L1^ΔWDR^ cells have lower LLP degradation capacity. (**A**,**B**) WT-HeLa cells were firstly labeled by AHA for 18 h, then short-lived proteins were chased for 2 h before cells were treated with 0.5 μM of Baf (Bafilomycin A1) or 1 μM of Torin1 with or without Baf and incubated in regular culture medium supplemented with 10× L-methionine for 12 h. Cells were collected and incubated with “Click” reaction mixture. The degradation of LLPs was analyzed by flow cytometry (**A**) and quantified (**B**) by relative fluorescence intensity per cell in (**A**), *n* = 3. * *p* < 0.05, *** *p* < 0.001, and **** *p* < 0.0001. (**C**–**H**) ATG16L1KO-HeLa cells (**C**,**D**), GFP-ATG16L1-HeLa cells (**E**,**F**), and GFP-ATG16L1^ΔWDR^-HeLa (**G**,**H**) were treated with 1 μM of Torin1 and were incubated in regular culture medium supplemented with 10× L-methionine for 12 h after labeling by AHA. Degradation of LLPs was analyzed by flow cytometry and quantified by relative fluorescence intensity per cell, *n* = 3. *** *p* < 0.001, **** *p* < 0.0001, ns: not significant. (**I**,**J**) WT-HeLa cells (WT), ATG16L1KO-HeLa (KO), GFP-ATG16L1-HeLa cells (FL), GFP-ATG16L1^ΔWDR^-HeLa cells (ΔWDR) were firstly labeled by AHA for 18 h, then short-lived proteins were chased for 2 h. Cells were incubated in regular culture medium supplemented with 10× L-methionine for 12 h. Degradation of basal LLPs was analyzed by flow cytometry and quantified, respectively, *n* = 3. *** *p* < 0.001, **** *p* < 0.0001, ns: not significant. (**K**,**L**) GFP-ATG16L1^ΔWDR^-HeLa cells and WT-HeLa cells were treated with 1 μM of Torin1 and 0.5 μM of Baf (Bafilomycin A1) for 3 h. Protein level of LC3 was analyzed by immunoblotting (**K**) and quantified (**I**), *n* = 3. * *p* < 0.001, **** *p* < 0.0001.

Bafilomycin A1 (Baf) is a widely used autophagic inhibitor that hinders fusion between autophagosomes and lysosomes and alkalizes the lysosomal lumen [45]. To verify the feasibility of this method, we administered Torin1 with or without Baf in WT-HeLa cells and measured the degradation levels of LLPs. As anticipated, the degradation level of LLPs significantly increased in the Torin1 group and decreased in the Baf group compared to control group (culture medium, CM). Furthermore, the degradation of Torin1-induced LLPs was significantly inhibited by Baf (Figure 3A,B), indicating the reliability of this approach. We then administered Torin1 to ATG16L1KO-HeLa, GFP-ATG16L1-HeLa, and GFP-ATG16L1^ΔWDR^-HeLa cells and observed that LLPs were not degraded in ATG16L1KO-HeLa cells, in which autophagy was completely blocked. However, Torin1 was still able to induce significant degradation of LLPs in both GFP-ATG16L1-HeLa and GFP-ATG16L1^ΔWDR^-HeLa cells (Figure 3C–H). This suggests that GFP-ATG16L1^ΔWDR^-HeLa cells are still capable of mediating Torin1-induced LLP degradation.

We further compared the degradation capacity of LLPs in WT-HeLa (WT), ATG16L1KO-HeLa (KO), GFP-ATG16L1-HeLa (full length, FL), and GFP-ATG16L1^ΔWDR^-HeLa (ΔWDR) cells under basal conditions without any treatment. No significant difference was observed between WT-HeLa (WT) and GFP-ATG16L1-HeLa (FL) cells regarding LLP degradation capacity. However, the level of LLP degradation in GFP-ATG16L1^ΔWDR^-HeLa (ΔWDR) cells was almost equivalent to that in ATG16L1KO-HeLa (KO) cells, which was significantly suppressed compared to WT-HeLa (WT) and GFP-ATG16L1-HeLa (FL) cells (Figure 3I,J). We subsequently detected LC3-II levels induced by Torin1 and Baf in WT-HeLa and GFP-ATG16L1^ΔWDR^-HeLa cells. The results showed that LC3-II could be induced in both cell types when treated with Torin1 or Baf. However, Baf induced a significant increase in LC3-II levels in WT-HeLa cells when compared to GFP-ATG16L1^ΔWDR^-HeLa cells (Figure 3K,L).

These results collectively indicate that the deletion of the WDR domain of ATG16L1 impairs the basal autophagic degradation capacity of cells.

### 2.3. WDR Deletion Does Not Affect the Lysosome Degradation Function and the Fusion between Autophagosome and Lysosome

**Figure 4:**
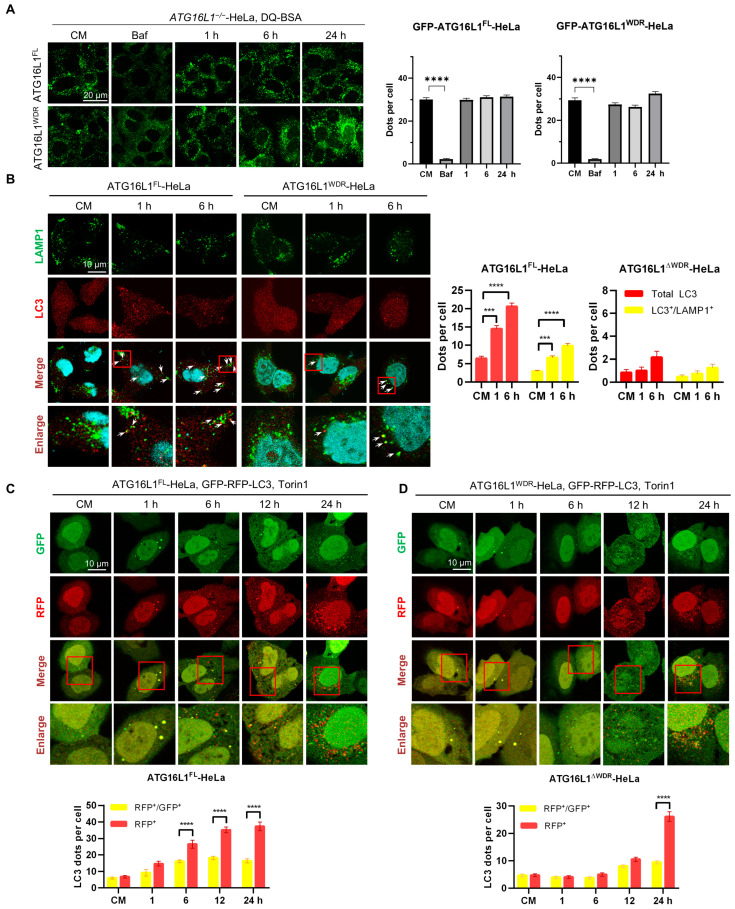
Deletion of WDR contributes to stalled autophagy flux without affecting fusion steps and lysosome function. (**A**) *ATG16L1^−/−^*-HeLa cells stably expressing GFP-ATG16L1^FL^ and GFP-ATG16L1^ΔWDR^ were preincubated with DQ-BSA Green working solution for 2 h followed by treatment with 0.5 μM of Bafilomycin A1 for 6 h and 4 μM of Torin1 for the indicated time. Confocal images were detected, scale bar = 20 μm. Dots per cell were quantified. **** *p* < 0.0001. (**B**) *ATG16L1^−/−^*-HeLa cells stably expressing GFP-ATG16L1^FL^ and GFP-ATG16L1^ΔWDR^ were treated with 4 μM of Torin1 for 1, 6 h and then stained for LC3 and LAMP1, scale bar = 10 μm. The red frames represent the area to be enlarged.Quantification of LC3 total dots (red) and partial colocalization with LAMP1 (yellow). *** *p* < 0.001, **** *p* < 0.0001. (**C**) *ATG16L1^−/−^*-HeLa cells expressed GFP-ATG16L1 ^FL^ were transiently transfected with GFP-RFP-LC3. After 24 h expression, the cells were then treated with 4 μM of Torin1 for 1, 6, 12, 24 h. LC3 dots were analyzed and quantified, scale bar = 10 μm. The red frames represent the area to be enlarged. **** *p* < 0.0001. (**D**) *ATG16L1^−/−^*-HeLa cells expressed GFP-ATG16L1^ΔWDR^ were transiently transfected with GFP-RFP-LC3. After 24 h expression, the cells were then treated with 4 μM of Torin1 for 1, 6, 12, 24 h. LC3 dots were analyzed and quantified, scale bar = 10 μm. The red frames represent the area to be enlarged. **** *p* < 0.0001.

Lysosomes are major organelles involved in protein degradation in acidic environments. As the degradation of p62 and LLPs was suppressed or delayed in ATG16L1^ΔWDR^-HeLa cells, we speculated whether the lysosomal degradation function or the fusion process between lysosomes and LC3-positive structures might be impaired. To determine this, we first measured lysosomal function using a DQ-BSA assay. Figure 4A shows that neither ATG16L1^FL^-HeLa nor ATG16L1^ΔWDR^-HeLa, treated with or without Torin1, suppressed lysosomal degradation function even after 24 h, except for the V-ATPase inhibitor Baf, which alkalized the lysosomal lumen. Subsequently, we analyzed the colocalization of LC3 and LAMP1 to observe the fusion between LC3-positive structures and lysosomes in ATG16L1^ΔWDR^ cells. The results showed that, in ATG16L1^FL^-HeLa cells treated with Torin1 for 1 h, the LC3-positive structures increased and overlapped with lysosomes (Figure 4B). In contrast, there was no significant increase in such overlap in ATG16L1^ΔWDR^-Hela cells, even after 6 h of treatment, due to the lower level of LC3 dots.

Next, we used a tandem LC3 assay to measure the fusion between lysosomes and LC3-positive structures. In ATG16L1^FL^-HeLa cells treated with Torin1, there was an increase in both red LC3 dots (RFP^+^) and yellow LC3 dots (GFP^+^RFP^+^), and the number of red dots was significantly higher than that of yellow dots, indicating that the fusion step was not affected (Figure 4C). However, this difference between the red and yellow dots in ATG16L1^ΔWDR^-Hela cells was not observed until 24 h of Torin1 treatment (Figure 4D), suggesting that the fusion step in ATG16L1^ΔWDR^-Hela cells was functional, but delayed when LC3 dots were induced.

Taken together, our results suggest that, although the accumulation of LC3-positive structures was delayed, the deletion of the WDR domain of ATG16L1 did not block lysosomal degradation or fusion between autophagosomes and lysosomes.

### 2.4. WDR Deletion Reduces the Interaction of ATG16L1 with FIP200 and WIPI2 

**Figure 5:**
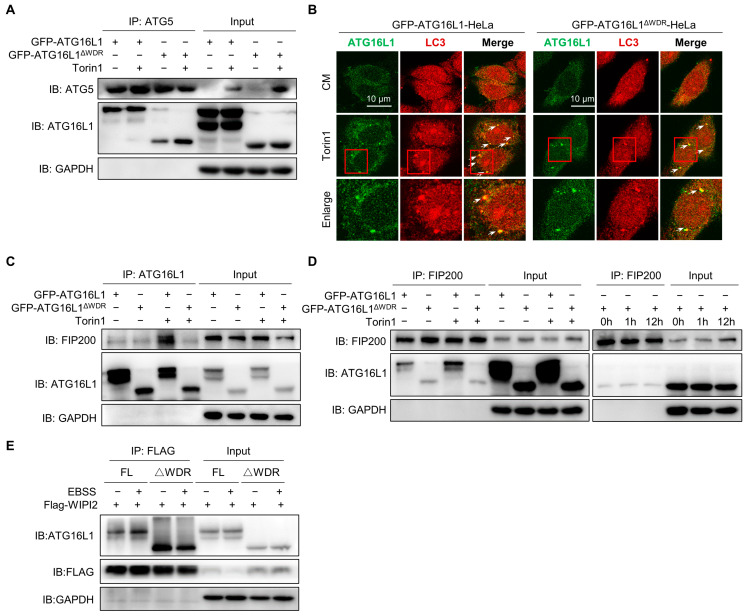
Deletion of WDR reduces the interaction of ATG16L1 with FIP200 and WIPI2b instead of the interaction of ATG16L1 with ATG5. (**A**) *ATG16L1^−/−^*-HeLa cells expressing GFP-ATG16L1/-ATG16L1^ΔWDR^ were treated with 1 μM of Torin1 for 1 h. Immunoprecipitation of ATG5 was performed in cell lysates; protein levels of ATG16L1 and ATG5 were analyzed. (**B**) *ATG16L1^−/−^*-HeLa cells stably expressing GFP-ATG16L1 and GFP-ATG16L1^ΔWDR^ were treated with 1 μM of Torin1 for 1 h and stained for LC3. Then, confocal images of LC3 and GFP-ATG16L1 and GFP-ATG16L1^ΔWDR^ were analyzed, scale bar = 10 μm. The red frames represent the area to be enlarged. (**C**,**D**) *ATG16L1^−/−^*-HeLa cells expressing GFP-ATG16L1 or GFP-ATG16L1^ΔWDR^ were treated with or without 1 μM of Torin1 for 1 h (C or the left of D) or 12 h, immunoprecipitation was performed using ATG16L1 antibody (**C**) and FIP200 antibody (**D**). Protein levels of FIP200 and ATG16L1 were analyzed by immunoblotting. (**E**) *ATG16L1^−/−^*-HeLa cells expressed GFP-ATG16L1 or GFP-ATG16L1^ΔWDR^ were treated with or without EBSS for 3 h. Immunoprecipitation of WIPI2-binding proteins from cell lysates was analyzed.

Previous results have shown that deletion of the WDR domain resulted in reduced degradation of p62 and LLP, without impairing lysosomal degradation function or the fusion process between lysosomes and LC3-positive structures. We were wondering if the upstream or downstream signals of ATG16L1 could affect the degradation level of canonical autophagy. The ATG12–ATG5–ATG16L1 complex could be formed by ATG16L1 binding with ATG5 to mediate LC3 lipidation as an E3-like ligase [46]. We observed a slightly weaker interaction between ATG16L1^ΔWDR^ and ATG5 compared to that of ATG16L1 at the basal level. However, after Torin1 treatment, the amount of ATG5 that precipitated with ATG16L1 and ATG16L1^ΔWDR^ remained nearly identical (Figure 5A). This result clearly suggested that the deletion of the WDR does not affect the interaction between ATG16L1 and ATG5-ATG12. Furthermore, deletion of the WDR domain did not affect the colocalization of LC3 with ATG16L1 truncates after Torin1 treatment (Figure 5B).

It has been reported that FIP200 could regulate late events of autophagosome formation through direct interaction with Atg16L1 [47]. FIP200 directly interacts with a short domain of ATG16L1 which was termed the FIP200-Binding Domain (FBD) [48]. This interaction between two upstream ATG complexes (ULK1 complex and Atg16L1 complex) regulates the stability of the ULK1 complex on one hand and enables the proper targeting of Atg16L1 to the isolation membrane on the other [47]. We performed immunoprecipitation of the binding proteins of ATG16L1 and FIP200 as indicated. The results showed that autophagic stimulation increased the interaction between full-length ATG16L1 and FIP200; however, those interactions of ATG16L1^ΔWDR^ remained unchanged (Figure 5C). Notably, the interaction between ATG16L1^ΔWDR^ and FIP200 remained weak even 12 h after Torin1 treatment (Figure 5D). Existing studies have demonstrated that WIPI2 directly interacts with ATG16L1 and is responsible for ATG16L1 complex recruitment to the forming autophagosome, then resulting in LC3 lipidation [49]. In this study, we observed that the interaction between ATG16L1^ΔWDR^ instead of ATG16L1 and WIPI2 did not alter after EBSS treatment (Figure 5E).

These results collectively indicate that, although the FIP200 and WIPI2 binding domains of ATG16L1 are intact, deletion of the WDR domain reduces the interaction of ATG16L1 with FIP200 and WIPI2, thereby contributing to impaired canonical autophagy in ATG16L1^ΔWDR^ cells.

### 2.5. Deletion of the WDR Domain or Mutation of K490A of ATG16L1 Inhibits Chemical-Induced NCA via Suppressing V-ATPase-ATG16L1 Axis

**Figure 6:**
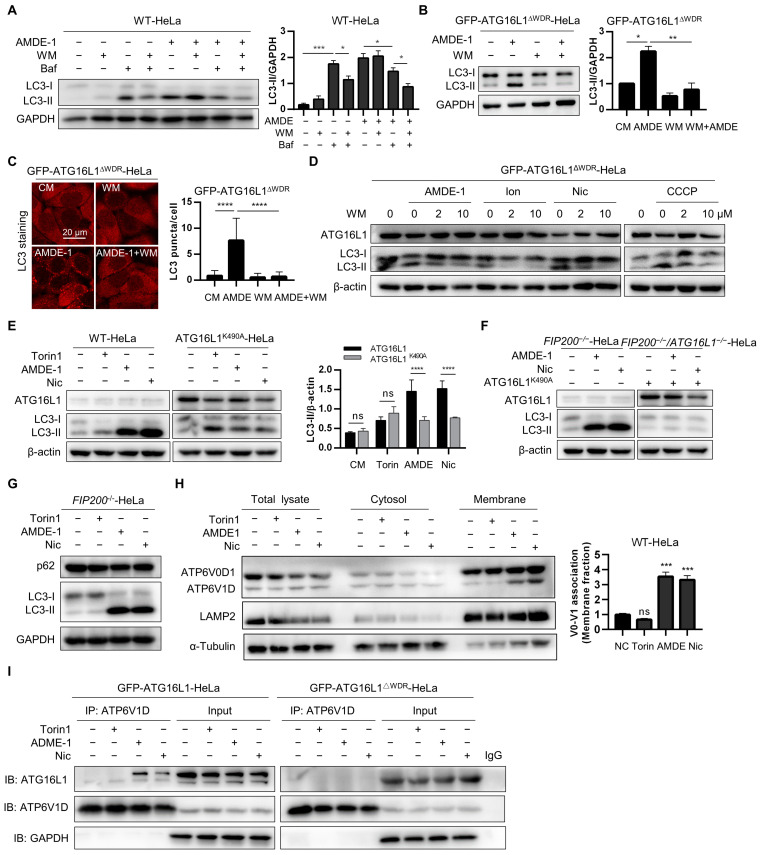
Chemical-induced NCA required the WDR domain and K490 of ATG16L1. (**A**) WT-HeLa cells treated with 10 μM of AMDE-1 with or without 2 μM of wortmannin (WM) or 1 μM of bafilomycin A1 (Baf) for 6 h. LC3 was analyzed and quantified, *n* = 3. * *p* < 0.05, *** *p* < 0.001, ns: not significant. (**B**) *ATG16L1^−/−^*-HeLa cells stably expressing GFP-ATG16L1^ΔWDR^ were treated with indicated 10 μM of AMDE-1 with or without 2 μM of WM for 6 h. Protein level of LC3-II was analyzed and quantified, *n* = 3. * *p* < 0.05, ** *p* < 0.01. (**C**) *ATG16L1^−/−^*-HeLa cells stably expressing GFP-ATG16L1^ΔWDR^ were treated with 10 μM of AMDE-1 with or without 2 μM of WM for 6 h. Endogenous LC3 was stained and analyzed, scale bar = 20 μm. LC3 puncta per cell were quantified, *n* = 50. **** *p* < 0.0001. (**D**) *ATG16L1^−/−^*-HeLa cells stably expressing GFP-ATG16L1^ΔWDR^ were treated with 10 μM of AMDE-1, 10 μM of Ion (ionomycin), 10 μM of Nic (niclosamide), 30 μM of CCCP with or without WM (2 or 10 μM), respectively, for 6 h. Protein levels of ATG16L1 and LC3 were analyzed. (**E**) WT-HeLa and ATG16L1^K490A^-HeLa cells were treated with 2 μM of Torin1, 10 μM of AMDE-1, and 10 μM of niclosamide (Nic) for 6 h. Immunoblot and quantification of ATG16L1 and LC3 were analyzed, *n* = 3. **** *p* < 0.0001, ns: not significant. (**F**) *FIP200^−/−^*-HeLa and *FIP200^−/−^/ATG16L1^−/−^*-HeLa cells stably expressing ATG16L1^K490A^ were treated with 10 μM of AMDE-1 and 10 μM of Nic for 6 h. Immunoblot of ATG16L1 and LC3 were analyzed. (**G**) *FIP200^−/−^*-HeLa cells were treated with 2 μM of Torin1, 10 μM of AMDE-1, and 10 μM of niclosamide (Nic) for 6 h. Immunoblot of LC3 was analyzed, *n* = 3. (**H**) WT-HeLa cells were treated with 2 μM of Torin1, 10 μM of AMDE-1, and 10 μM of niclosamide (Nic) for 6 h. After fractionation, total lysate, cytosol, and membrane fractions were subjected to Western blotting. ATP6V0D1 and ATP6V1D were analyzed and V0–V1 association of membrane fraction were quantified, *n* = 3. *** *p* < 0.001, ns: not significant. (**I**) *ATG16L1^−/−^*-HeLa cells expressing GFP-ATG16L1 or GFP-ATG16L1^ΔWDR^ were treated with 2 μM of Torin1, 10 μM of AMDE-1, and 10 μM of niclosamide (Nic) for 6 h. Immunoprecipitation of ATP6V1D was performed in cell lysates; protein levels of ATG16L1 and ATP6V1D were analyzed.

AMDE-1 was initially discovered to initiate canonical phagophore formation through the AMPK-mTOR signaling pathway and to inhibit lysosomal function and autophagy flux in WT cells. It was subsequently found to induce NCA in FIP200-deficient cells [25,50]. However, the mechanism by which AMDE-1 induces NCA remains unclear. To verify the regulatory roles of AMDE-1 in initiating canonical autophagy and inducing NCA, WT-HeLa cells were treated with AMDE-1 with or without wortmannin (WM) or Baf. The results showed that WM significantly suppressed the accumulation of LC3-II caused by Baf, but not by AMDE-1 (Figure 6A). These data suggested that, although both Baf and AMDE-1 could inhibit autophagy flux in WT cells, most of the LC3-II accumulation caused by Baf is likely due to the canonical autophagy pathway. Conversely, LC3-II accumulation induced by AMDE-1-induced NCA remained largely unaffected by the canonical autophagy inhibitor WM. Furthermore, both immunoblotting and immunofluorescence analyses of LC3 showed that AMDE-1-induced LC3-II could be significantly inhibited by WM in NCA-deficient GFP-ATG16L1^ΔWDR^-HeLa cells (Figure 6B,C). We then administered WM in GFP-ATG16L1^ΔWDR^-HeLa cells and confirmed that LC3-II induced by chemicals such as ionomycin, niclosamide, and CCCP, which have been identified to regulate both canonical autophagy and NCA in WT cells [24,44], could be strongly reduced by WM (Figure 6D). These results further indicated that WDR is required for NCA induced by the chemicals we examined, which is consistent with the existing reports on other NCA or CASM [37,42].

The K490 residue of ATG16L1 located on the WDR domain has been shown to play a key role in LAP induced by pathogenic bacteria [42]. In this study, canonical autophagy inducer Torin1 and bifunctional autophagy inducers AMDE-1 and niclosamide were administered to WT-HeLa cells and *ATG16L1^−/−^*-HeLa cells stably expressing ATG16L1^K490A^, respectively. We found that the LC3 lipidation induced by Torin1 was not significantly different between WT-HeLa and ATG16L1^K490A^-Hela cells due to its canonical autophagy induction (Figure 6E), suggesting that the K490 residue is dispensable for canonical autophagy, which is consistent with a previous study [42]. However, AMDE-1 and niclosamide were found to significantly repress the ability to induce LC3 lipidation in ATG16L1^K490A^-HeLa cells compared to WT-Hela cells (Figure 6E), indicating that the NCA pathway was partially inhibited in ATG16L1^K490A^-HeLa cells. Subsequently, *FIP200^−/−^*-HeLa cells, in which only NCA can be induced, and FIP200/ATG16L1 double knockout HeLa cells stably expressing ATG16L1^K490A^ were treated with AMDE-1 and niclosamide, respectively (Figure 6F,G). Notably, both AMDE-1 and niclosamide were able to increase LC3-II levels in *FIP200^−/−^*-HeLa cells, even with ATG16L1^K490A^ overexpression (Figure 6F). These data suggest that the chemical-induced NCA is dependent on the K490 residue of ATG16L1.

Existing studies have demonstrated that the V-ATPase-ATG16L1 axis is necessary for noncanonical LC3 lipidation, and V-ATPase serves as a universal regulator of LAP and NCA [41,51]. The cytosolic V1 sector is associated with the membrane-bound V0 sector to form a functional holoenzyme of V-ATPase [52]. An increased V0–V1 association can drive NCA, xenophagy, and V-ATPase–ATG16L1 interaction [41,43]. Therefore, we examined the effects of the chemicals we used and found that both AMDE-1 and niclosamide, rather than Torin1, significantly enhanced the association of V0–V1 in the membrane fraction (Figure 6H). Furthermore, we assessed the interaction between ATG16L1 and V-ATPase and found that AMDE-1 and niclosamide could promote ATG16L1–ATP6V1D interaction, which was markedly stronger than Torin1 in *ATG16L1^−/−^*-Hela cells stably expressing GFP-ATG16L1. However, no such difference was found in *ATG16L1^−/−^*-Hela cells stably expressing ATG16L1^ΔWDR^, both before and after treatment with NCA inducers (Figure 6I).

Taken together, our results suggest that the deletion of the WDR domain or mutation of K490A of ATG16L1 could inhibit chemical-induced NCA by suppressing the V-ATPase-ATG16L1 axis.

### 2.6. Intact ATG16L1 with a Full-Length WDR Is Required for Canonical Autophagy and NCA

**Figure 7:**
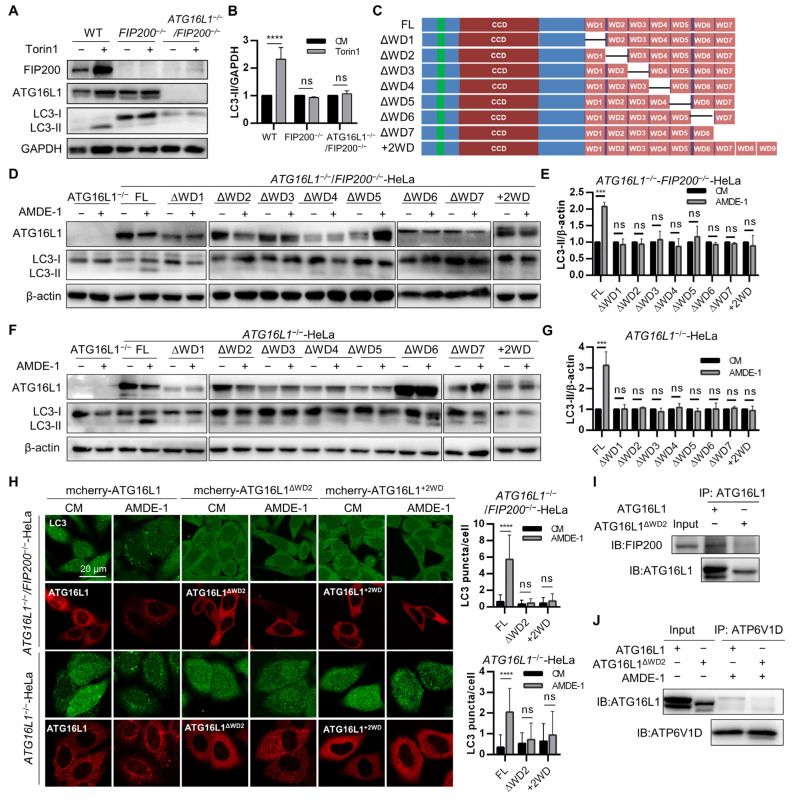
ATG16L1 with an incomplete WDR could not mediate canonical autophagy or NCA/CASM. (**A**,**B**) WT-HeLa, *FIP200^−/−^*-HeLa, and *ATG16L1^−/−^*-*FIP200^−/−^*-HeLa cells were treated with 1 μM of Torin1 for 6 h. FIP200, ATG16L1, and LC3 were analyzed and quantified, *n* = 3. **** *p* < 0.0001, ns: not significant. (**C**) Diagram of mutants with truncation/redundancy of WD40 fragments. (**D**,**G**) *ATG16L1^−/−^*/*FIP200^−/−^*-HeLa (**D**,**E**) or *ATG16L1^−/−^*-HeLa (**F**,**G**) cells were transiently transfected with ATG16L1 mutants for 42 h and then treated with 10 μM of AMDE-1 for 6 h. Protein levels of ATG16L1 and LC3 were analyzed and quantified, *n* = 3. *** *p* < 0.001, ns: not significant. (**H**) *ATG16L1^−/−^*/*FIP200^−/−^*-HeLa or *ATG16L1^−/−^*-HeLa cells were transiently transfected with ATG16L1 mutants for 42 h and then treated with 10 μM of AMDE-1 for 6 h. Cells were stained for LC3. The confocal images of mcherry-ATG16L1 mutants and LC3 were observed, scale bar = 10 μm. LC3 puncta per cell in (**H**) were quantified, *n* = 50. **** *p* < 0.0001, ns: not significant. (**I**) Immunoprecipitation using ATG16L1 antibody was performed in cell lysates of *ATG16L1^−/−^*-HeLa cells expressing GFP-ATG16L1, ATG16L1^ΔWD2^, and ATG16L1^ΔWDR^. Protein levels of FIP200 and ATG16L1 were analyzed. (**J**) *ATG16L1^−/−^*-HeLa cells expressing GFP-ATG16L1 and ATG16L1^ΔWD2^ were treated with 20 μM of AMDE-1 for 3 h. Immunoprecipitation of ATP6V1D was performed in cell lysates; protein levels of ATG16L1 and ATP6V1D were analyzed.

We have already demonstrated that the WDR domain is crucial for both canonical autophagy and chemical-induced NCA. The WDR domain consists of seven WD40 repeats and is almost half of the length of ATG16L1 [37]. To further explore which of these seven WD40 repeats is pivotal for NCA, we prepared *FIP200^−/−^*-HeLa cells and *ATG16L1^−/−^FIP200^−/−^* double knockout HeLa cells, which were resistant to Torin1-induced canonical autophagy (Figure 7A,B). We also constructed plasmids containing ATG16L1 mutants with different WD40 fragment truncations or two additional WD40 fragments to investigate their roles in LC3 lipidation (Figure 7C). 

FIP200 is a crucial protein of the ULK complex for initiating canonical autophagy. To investigate the role of pivotal WD40 fragments in mediating chemical-induced NCA, *ATG16L1^−/−^FIP200^−/−^* double knockout HeLa cells were transiently transfected with full-length ATG16L1, and its mutants followed by treatment with AMDE-1. Immunoblotting results showed that only full-length ATG16L1, rather than its mutants, could mediate AMDE-1-induced LC3 lipidation (Figure 7D,E). Similarly, only full-length ATG16L1 could mediate LC3 lipidation induced by niclosamide and CCCP (Figure S2A,C). Immunostaining results for LC3 confirmed these findings (Figure 7H), suggesting that chemical-induced NCA requires full-length ATG16L1.

We demonstrated that ATG16L1^ΔWDR^ can mediate weaker canonical autophagy, whereas ATG16L1 with an incomplete WDR cannot mediate NCA. Therefore, we transiently transfected ATG16L1 and its mutants into *ATG16L1^−/−^*-HeLa cells to explore the essential WD40 fragments mediating canonical autophagy. Immunoblotting results showed that only full-length ATG16L1, rather than its mutants, could mediate canonical autophagic LC3 lipidation induced by AMDE-1, niclosamide, Torin1, rapamycin, and amino acid starvation (Figure 7F,G and Figure S2B,D). Similar results were obtained using immunostaining for LC3 (Figure 7H and Figure S3A). It has been reported that overexpression of ATG16L1 can inhibit autophagy [46], and ATG16L1 may be unsuitable for transient transfection to study its function [53]. To avoid false-negative results, *ATG16L1^−/−^*-HeLa cells stably expressing GFP-ATG16L1, GFP-ATG16L1^ΔWDR^, and GFP-ATG16L1^ΔWD2^ were prepared and treated with autophagy inducers (EBSS, rapamycin, Torin1) and autophagy blockers (chloroquine). Immunoblotting of LC3 showed that full-length ATG16L1 and ATG16L1^ΔWDR^, rather than ATG16L1^ΔWD2^, could mediate canonical autophagic LC3 lipidation (Figure S2E), suggesting that ATG16L1 with an incomplete WDR cannot mediate canonical autophagy.

We sought to investigate why ATG16L1 with an incomplete WDR could not mediate canonical autophagy and chemical-induced NCA, and sought to elucidate this phenomenon through the interaction of ATG16L1 with FIP200. It is well established that ATG16L1 can directly bind with FIP200, which is essential for the initiation step of canonical autophagy [48]. Immunoprecipitation of ATG16L1 from GFP-ATG16L1-HeLa and GFP-ATG16L1^ΔWD2^-HeLa cells after 1 h of starvation revealed that full-length ATG16L1 could bind to FIP200, while ATG16L1^ΔWD2^ could not (Figure 7I), suggesting that the absence of a specific WD40 segment prevents ATG16L1 from binding with FIP200 to mediate canonical autophagy. Furthermore, a study has shown that, in LAP, ATG16L1 can be recruited to bacteria-containing vesicles by V-ATPase via its interaction with the WDR domain to mediate LC3 lipidation [43]. The ATP6V1D immunoprecipitation assay, using GFP-ATG16L1-HeLa and GFP-ATG16L1^ΔWD2^-HeLa cells after AMDE-1 treatment, suggested that full-length ATG16L1 had a stronger binding affinity with ATP6V1D than ATG16L1^ΔWD2^ (Figure 7J).

These data indicated that ATG16L1 with an incomplete WDR cannot mediate NCA due to its weak interaction with V-ATPase subunits.

## 3. Discussion

Existing studies have shown that deficiency of the WDR domain of ATG16L1 can still mediate LC3 lipidation and LLP degradation in canonical autophagy [42,54]. Therefore, the WDR domain of ATG16L1 might not be necessary for canonical autophagy. However, mice with WDR-deficient ATG16L1 exhibited symptoms of neurodegenerative disease [40], which is commonly caused by autophagy blockade [55]. Whether the WDR domain of ATG16L1 is dispensable for canonical autophagy remains unclear. In this study, contrary to previous studies, we demonstrated that ATG16L1 plays an important role in both canonical autophagy and chemical-induced NCA. The ATG16L1 with an incomplete WDR domain loses its ability to mediate the aforementioned two types of autophagy. 

Autophagy flow in *ATG16L1*^−/−^-HeLa cells stably expressing full-length ATG16L1 with or without an intact WDR domain was detected under treatment with the mTOR inhibitor Torin1 or amino acid starvation. The LC3 lipidation mediated by full-length ATG16L1 was accumulated faster and stronger than that mediated by ATG16L1^ΔWDR^. The degradation levels of LLPs in different HeLa cells were also measured. Although the LLP degradation in ATG16L1^ΔWDR^-HeLa cells increased similarly to Torin1 stimulation, the basal LLP degradation capacity in ATG16L1^ΔWDR^ cells was significantly blocked, similar to that in ATG16L1 knockout cells, compared to cells with full-length ATG16L1. Our data further confirmed that ATG16L1, lacking the WDR domain, can mediate canonical autophagy after stimulation by canonical autophagy inducers, albeit with reduced ability, possibly due to the lack of a WDR domain. Consequently, basal autophagy was strongly blocked, and autophagic flux lagged. Detailed analysis of the upstream and downstream signals of ATG16L1 clearly demonstrated that the deletion of the WDR domain, before or after chemical treatments, significantly weakened the interaction of ATG16L1 with FIP200 and WIPI2b without affecting the interaction between ATG16L1 and ATG5. This alteration appears to be the main reason why canonical autophagy lagged in the ATG16L1^ΔWDR^-Hela cells. 

p62 is among the most common receptors in canonical autophagy and is responsible for protein recruitment for degradation through the autophagy pathway. As anticipated, the protein level of p62 in cells with full-length ATG16L1 significantly decreased upon activation of canonical autophagy [56]. However, p62 failed to degrade and remained unchanged within 12 h of starvation when the WDR domain was deleted, probably because of weaker basal autophagy levels. 

ATG16L1 is a key protein in both canonical autophagy and NCA, and its WDR domain is essential for monensin-induced LAP and NCA [42]. The present study showed that chemical-induced NCA depends on the WDR domain of ATG16L1 by employing a cell model with an artificial WDR deletion. Bifunctional autophagy inducers like AMDE-1, niclosamide, CCCP, and sodium oleate could induce NCA in a WDR domain-dependent manner [24,25,44,57], indicating that the WDR domain is ubiquitously required for chemical-induced NCA.

The WDR domain of ATG16L1 accounts for about half of the full length of ATG16L1 and comprises seven WD40 repeat fragments. Our results showed that only full-length ATG16L1 could mediate noncanonical LC3 lipidation; deletion or addition of any WD40 fragment would make ATG16L1 incapable of mediating NCA completely. Interestingly, ATG16L1 with or without the WDR domain can both mediate canonical autophagy, albeit autophagy activity decreased following WDR domain deletion. However, ATG16L1 with an incomplete WDR could not mediate the LC3 lipidation by canonical autophagy inducers. The ATG16L1 contains seven WD40 repeat fragments arranged as a seven-membered ring [37]. Based on this, we hypothesized that for canonical autophagy, partial deletion or addition of WD40 fragments would destroy the seven-membered ring structure, thereby increasing the steric hindrance. For NCA, the seven-membered ring structure of the WDR may be essential for binding with other key proteins. The V-ATPase was reported as a key protein to recruit ATG16L1 via its WDR domain, and the V-ATPase–ATG16L1 interaction could be driven by the V0–V1 association [41,43]. Our study also showed that the chemicals we used significantly increased the association between V0–V1 and the V-ATPase–ATG16L1 interaction; however, WDR domain deletion reduced the interaction between ATG16L1 and V1 subunit of V-ATPase. Furthermore, studies have shown that K490 residue at the WDR domain of ATG16L1 is necessary for LC3 lipidation in LAP [42,58]. Similarly, K490 also played a key role in our chemical-induced NCA rather than canonical autophagy, further suggesting that the structural integrity of the WDR domain plays a decisive role in the function of the whole protein. However, whether other proteins were involved in the dysfunction of canonical autophagy and NCA mediated by ATG16L1 with an incomplete WDR domain requires further research.

Compounds that have previously been identified as NCA inducers can also induce canonical autophagy in WT cells [50,59]. We found that the level of NCA in WT cells in which different types of autophagy occurred simultaneously was significantly lower compared to FIP200-deficient cells, indicating that the canonical autophagy and NCA pathways might maintain a certain balance during treatment with bifunctional compounds in WT cells.

In summary, our study demonstrated that only ATG16L1 with an intact WDR domain can successfully mediate both canonical autophagy and chemical-induced NCA/CASM. This work will provide further understanding of the multifaceted role of ATG16L1 in canonical autophagy and NCA/CASM and help differentiate chemical-induced canonical autophagy from NCA mediated by ATG16L1 from different perspectives.

## 4. Materials and Methods

### 4.1. Antibodies and Reagents

Antibodies for immunoblotting: anti-ATG16L1 (M150-3), anti-p62 (PM045), anti-Flag (M185-3L) were purchased from Medical & Biological Laboratories (Minato-ku, Tokyo, Japan); anti-LC3B (L7543), and anti-β-actin (A1978) were purchased from Sigma Aldrich (Lovonia, MI); anti-FIP200 (17250-1-AP), anti-ATP6V1D (14920-1-AP), and anti-GAPDH (60004-1) were purchased from Proteintech (Rosemont, IL, USA); anti-LAMP1 (H4A3) was purchased from Developmental Studies Hybridoma Bank (Iowa City, IA, USA); HRP-conjugated goat anti-mouse IgG (SA00001-1) and HRP-conjugated goat anti-rabbit IgG antibody (SA00001-2) were purchased from Proteintech (Rosemont, IL, USA). Antibodies for immunofluorescence: anti-LC3B (PM036) and anti-p62 were purchased from Medical & Biological Laboratories (Minato-ku, Tokyo, Japan); anti-GFP (sc-9996) was purchased from Santa Cruz Biotechnology (Paso Robles, CA, USA). Alexa Fluor 594 (A11012), Alexa Fluor 488 (A32731), and DQ-BSA Green (D-12050) were purchased from Thermo Fisher Scientific (Waltham, MA, USA). 

Chemicals: AMDE-1 (4N-049) was purchased from Bionet (Seoul, Republic of Korea); niclosamide (481909) and CCCP (555-60-2) were purchased from Calbiochem (Darmstadt, Germany); Bafilomycin A1 (MB5505) and sodium oleate (MB2952) were purchased from Meilunbio (Dalian, China); Torin1 (475991) was purchased from Sigma-Aldrich (Lovonia, MI, USA); Puromycin (S7417) was from Selleckchem (Shanghai, China); Chloroquine (CQ, A506569) was purchased from Sangon Biotech (Shanghai, China); Wortmannin (W-2990) and rapamycin (R-5000) were purchased from LC Laboratories (New Boston, TX, USA); Ionomycin (GC15446) was purchased from GLPBIO (Montclair, CA, USA). 

Reagents: Dulbecco’s modified Eagle’s medium (DMEM, 11965092) was purchased from Thermo Scientific (Waltham, MA, USA); Fetal bovine serum (FBS) (F8318) was purchased from Sigma (Livonia, MI, USA); Liopfectamine 2000 (11668-027) was from Invitrogen (Carlabad, CA, USA); Opti-MEM (31985-070) was from Gibco (Carlabad, CA, USA); Glucose (A100188-0500), CuSO_4_ (C3008), D-mannitol (MB0335), Sucrose (SB0498), and DTT (A100281-0005) were from Sangon Biotech (Shanghai, China); 5% goat serum (AR0009) was from Boster Biological Technology (Wuhan, China); Protease inhibitor cocktail (A32961) and protein A/G immunomagnetic beads (B23202) were from Bimake (Houston, TX, USA); Trypsinization (25200056) and L-methionine-, L-cystatin-, pyruvate-, and glutamine-free DMEM (21013024) were from Thermo Fisher Scientific (Waltham, MA, USA); EDTA (P0013B) was from Beyotime (Hangzhou, China); HEPES-KOH (BB21011) was purchased from BestBio (Shanghai, China); Fast Mutagenesis System (FM111-01) was from TransGen Biotech (Beijing, China); Glutamine (GB0224) was from BBI Life Sciences (Shanghai, China); L-Cystine (C7480) was from Solarbio (Beijing, China); Sodium pyruvate (S817535) was from Macklin (Shanghai, China); Dialyzed FBS (2148391) was from Biological Industries (Kibbutz Beit-Haemek, Israel); AHA (HY-140346A) was from MCE (Jersey, NJ, USA); L-methionine (M101131) was from Aladdin (Shanghai, China); TAMRA alkyne (AAT-487) was from AAT Bioquest (Sunnyvale, CA, USA); TCEP (580560) was from Calbiochem (Darmstadt, Germany); TBTA (GC45003) was from GLPBIO (Montclair, CA, USA); polybrene (40804ES76) was purchased from Yeasen (Shanghai, China); ECL Western blotting substrate (180-5001) was purchased from Tanon (Shanghai, China).

### 4.2. Cell Lines and Culture Conditions

HeLa cells and HEK-293T cells (ATCC, CRL-1573) were cultured in DMEM with 10% FBS at 37 °C, 5% (*v*/*v*) CO_2_ incubator. 

WT-HeLa and *ATG16L1^−/−^*-HeLa cells were purchased from Abclonal Technology Co., Ltd. (Wuhan, China). *FIP200*^−/−^-HeLa and *ATG16L1*^−/−^-*FIP200*^−/−^-HeLa cells were constructed by our laboratory using CRISPR/Cas9 system (FIP200-sgRNA: AAGATTGCTATTCAACACC). *ATG16L1*^−/−^-HeLa cells stably expressing GFP-ATG16L1, GFP-ATG16L1^ΔWDR^, GFP-ATG16L1^1−230^, ATG16L1^K490A^, GFP-ATG16L1^ΔWD2^, and *ATG16L1*^−/−^-*FIP200*^−/−^-HeLa cells stably expressing GFP-ATG16L1, GFP-ATG16L1^ΔWDR^, ATG16L1^K490A^, GFP-ATG16L1^ΔWD2^ were created by our laboratory using lentiviral particles.

Lentiviral particles were acquired by transfecting lentiviral plasmids harboring the desired gene into HEK293T cells together with psPAX2 (12259, Addgene, Watertown, MA, USA) and pMD2G (12260, Addgene) at a ratio of 3:2:1, respectively. The supernatants were collected 48 h later after transfection and filtered through a 0.45 μM filter membrane. *ATG16L1^−/−^*-HeLa cells and *ATG16L1^−/−^*-*FIP200^−/−^*-HeLa cells were transfected with lentivirus for 48 h with 10 µg/mL of polybrene. Then, cells were selected with 5 µg/mL of puromycin or by flow cytometry for stable cell lines.

### 4.3. Plasmid Construction

For the construction of plasmid-harbored ATG16L1 and its mutants with WD40 fragment deletion or addition, ATG16L1 was cloned from HEK293 and ATG16L1 with partial deletion of the WDR domain or partial addition of the WDR domain was cloned by overlapping extended PCR. Due to the limitation of the overlapping extension PCR, the missing amino acids of ATG16L1^ΔWD1^ in this study were residues 317–359, the missing amino acids of ATG16L1^ΔWD2^–ATG16L1^ΔWD7^ were consistent with those in the Uniprot database, and two WD40 repeats added to the C-terminal of ATG16L1 were cloned from the protein WDR33 (details of vectors and primers are listed in Table S1, which can be found in Supplemental Materials).

For the construction of ATG16L1^K490A^, Fast Mutagenesis System was used. Primers were designed to mutate number 1468 and number 1469 bases from AA to GC (forward primer 5′-3′: GAGATGGAGCTGTTGGGAGCGATTACTGCC; reverse primer 5′-3′: GTATCAAGCTCTCTACCTCGACAACCCTCG) according to the protocol of kit. Plasmid-harbored ATG16L1 was used as template for PCR amplification; after PCR, plasmid-harbored ATG16L1^K490A^ was digested by DMT.

### 4.4. Plasmid Transfection

For 12-well plates, when the cell confluency reached 50%, HeLa cells were transfected using Lipo-2000 at 2 µL per 1 µg of plasmid, and 1 µg of plasmid per 1 mL of growth medium. An amount of 1 µg of plasmid was diluted in 100 µL of Opti-MEM without supplements and 2 µL of transfection reagent was diluted in another 100 µL of Opti-MEM without supplements for 5 min, then they were mixed together and incubated for 15 min at RT, and then added directly to cells grown in 800 µL of DMEM. Cells were returned to incubator and medium was replaced by 1 mL of complete DMEM after 6 h, then cells were incubated for 42 h.

### 4.5. DQ-BSA Assay

Cells were seeded in glass-bottomed dishes and incubated at 37 °C, 5% CO_2_, until they reached 60–70% confluency. DQ-BSA Green (Invitrogen, D12050) was diluted with EBSS (Meilunbio, MA0031) to a concentration of 10 µg/mL as working solution. After incubating with working solution preheated to 37 °C for 2 h, the cells were then washed twice with PBS and treated with drugs for 6 h at 37 °C, 5% CO_2_. Following treatments, cells were rinsed twice with PBS and then incubated with 4% paraformaldehyde for 30 min. Then, stained cells were washed three times with PBS and fluorescence was visualized by Confocal Laser Scanning Microscope (Olympus, FV3000, Tokyo, Japan).

### 4.6. Immunostaining

HeLa cells were rinsed briefly with PBS twice and fixed with 4% paraformaldehyde for 20 min. Then, cells were rinsed briefly with PBS twice and permeabilized with 0.3% TritonX-100 for 20 min at RT. After washing twice with PBS, permeabilized cells were blocked in 5% goat serum for 1 h at RT, incubated with primary antibodies overnight at 4 °C and incubated with Alex Fluor secondary antibodies for another 1 h at RT. Then, stained cells were washed three times with PBS and fluorescence was visualized by Confocal Laser Scanning Microscope (Olympus, FV3000) or EVOS FL Auto Cell Imaging System (Thermo Fisher Scientific). The confocal images for each experiment were captured at the same magnification; therefore, scale bars have only been included in the first image of each experiment.

### 4.7. Immunoblotting

Cells were lysed using RIPA buffer (50 mM Tris, pH 7.4, 150 mM NaCl, 1% TritonX-100, 0.1% SDS, 5% glycerol, 1 mM EDTA containing protease inhibitor cocktail) on ice for 15 min. Then, the cells’ lysates were separated from precipitate by centrifuging at 12,000× *g* for 15 min at 4 °C. The supernatant was quantified using BCA quantitative kit and denatured by boiling for 5 min. Immunoblotting was performed by loading the samples on SDS-PAGE gels, conducting electrophoresis, transferring the proteins to PVDF membranes (Millipore, ISEQ00010, Burlington, MA, USA), blocking the membranes with 5% skim milk for 1 h at RT, and then incubating the indicated antibodies for at least 12 h at 4 °C and HRP antibodies for 1 h at RT. Then, all PVDF membranes were incubated with ECL Western blotting substrate and protein signal was detected by Tanon 5200 Chemiluminescence Image analysis system (Tanon, 5200).

### 4.8. Immunoprecipitation Assay

HeLa cells were lysed on ice in IP lysis buffer (50 mM Tris-HCl, pH 7.4, 1% TritonX-100, 150 mM NaCl, 1 mM EDTA containing protease inhibitor cocktail) for 30 min. The lysed cells were pelleted at 12,000× *g* at 4 °C for 15 min and supernatants containing 1 mg of proteins were incubated with antibody overnight at 4 °C to form antigen–antibody complex. An amount 30 μL of protein A/G immunomagnetic beads was washed twice with binding buffer (50 mM Tris, 150 mM NaCl, 0.1% TritonX-100, pH 7.5) and incubated with antigen–antibody complex for 1 h at RT. Then, the beads were washed three times in washing buffer (50 mM Tris, 150 mM NaCl, 0.1% TritonX-100, pH 7.5) and proteins were eluted with 30 μL of 1× loading buffer at 95 °C for 5 min. Samples were analyzed by immunoblotting assay.

### 4.9. LLP Degradation Assay

LLP degradation assay was performed in accordance with the protocol reported before [60]. Cells were plated in 6-well plates and cultured in regular growth medium (DMEM supplemented with 10% FBS), and then incubated in a 5% CO_2_ atmosphere at 37 °C until the cells reached 80–90% confluency. Cells were washed twice in warmed PBS, and then incubated with 2 mL of L-methionine-free DMEM (L-methionine-, L-cystatin-, pyruvate-, and glutamine-free DMEM supplemented with 4 mM glutamine, 0.2 mM L-Cystine, 1 mM sodium pyruvate with 10% dialyzed FBS) for 30 min at 37 °C, 5% CO_2_. After incubation, the medium was replaced with L-methionine-free DMEM with 10% dialyzed FBS and 50 μM of AHA. After incubation for 18 h at 37 °C, 5% CO_2_, culture medium was replaced with regular medium with 10× L-methionine and incubated at 37 °C, 5% CO_2_ for 2 h. Then, cells were washed twice in warmed PBS and 2 mL regular medium with 10× L-methionine was added, and cells were treated with drugs for 12 h at 37 °C, 5% CO_2_. Cells were washed twice in warm PBS and collected to a 1.5 mL Eppendorf tube by trypsinization. Then, cells were washed once in cold PBS and fixed by 1 mL of 4% paraformaldehyde for 15 min at RT. Cells were washed once with 1 mL of 3% BSA in PBS and cold PBS, respectively, followed by permeabilization with 0.5 mL of 0.5% TritonX-100 in PBS for 20 min at RT. After permeabilization, cells were washed once with cold-PBS and 1 mL of 3% BSA in PBS, then cells were washed once more with cold PBS and incubated in the click reaction master mix (50 μM of TAMRA alkyne, 1 mM TCEP, 100 μM of TBTA, 1 mM CuSO_4_ in PBS) in the dark for 2 h at RT. After click reaction, cells were centrifuged at 1500 rpm at 4 °C for 10 min to remove the reaction buffer, then cells were washed once with 3% BSA in PBS and twice with cold PBS. Finally, cells were resuspended in 500 μL of PBS and analyzed by flow cytometry, then the relative intensity was calculated as reported.

### 4.10. Statistical Analysis

GraphPad prism 8.2.1 was used for statistical analysis. Student’s *t*-test (two-tailed, unpaired) was used to compare data of two groups, while one-way ANOVA was used to compare three or more groups. All data were obtained from at least three independent experiments and expressed as the mean ± SD. * *p* < 0.05, ** *p* < 0.01, *** *p* < 0.001, and **** *p* < 0.0001, ns: not significant. 

## Data Availability

The data that support the findings of this study are available from the corresponding author upon reasonable request.

## Supplementary Materials

The following supporting information can be downloaded at: https://www.mdpi.com/article/10.3390/ijms25084493/s1.

## Abbreviations

AD: Alzheimer’s disease, ATG: autophagy-related gene, CASM: conjugation of ATG8s to single membranes, Cul-3: Cullin-3, DMEM: Dulbecco’s modified Eagle’s medium, EBSS: Earle’s Balanced salt solution, FBS: Fetal bovine serum, LANDO: LC3-associated endocytosis, LAP: LC3-associated phagocytosis, LC3: microtubule-associated protein 1 light chain 3, LLPs: long-lived proteins, NCA: noncanonical autophagy, WDR: WD40 repeats, WM: Wortmannin.
