# Supplementary material for: Missing WD40 Repeats in ATG16L1 Delays Canonical Autophagy and Inhibits Noncanonical Autophagy
Source: Int J Mol Sci. 2024 Apr 19;25(8):4493. doi: 10.3390/ijms25084493 (PMC11050548; doi:10.3390/ijms25084493)
Supplement: Supplementary file 1 [file ijms-25-04493-s001.zip › ijms-2937743-supplementary.pdf]

Supplemental data

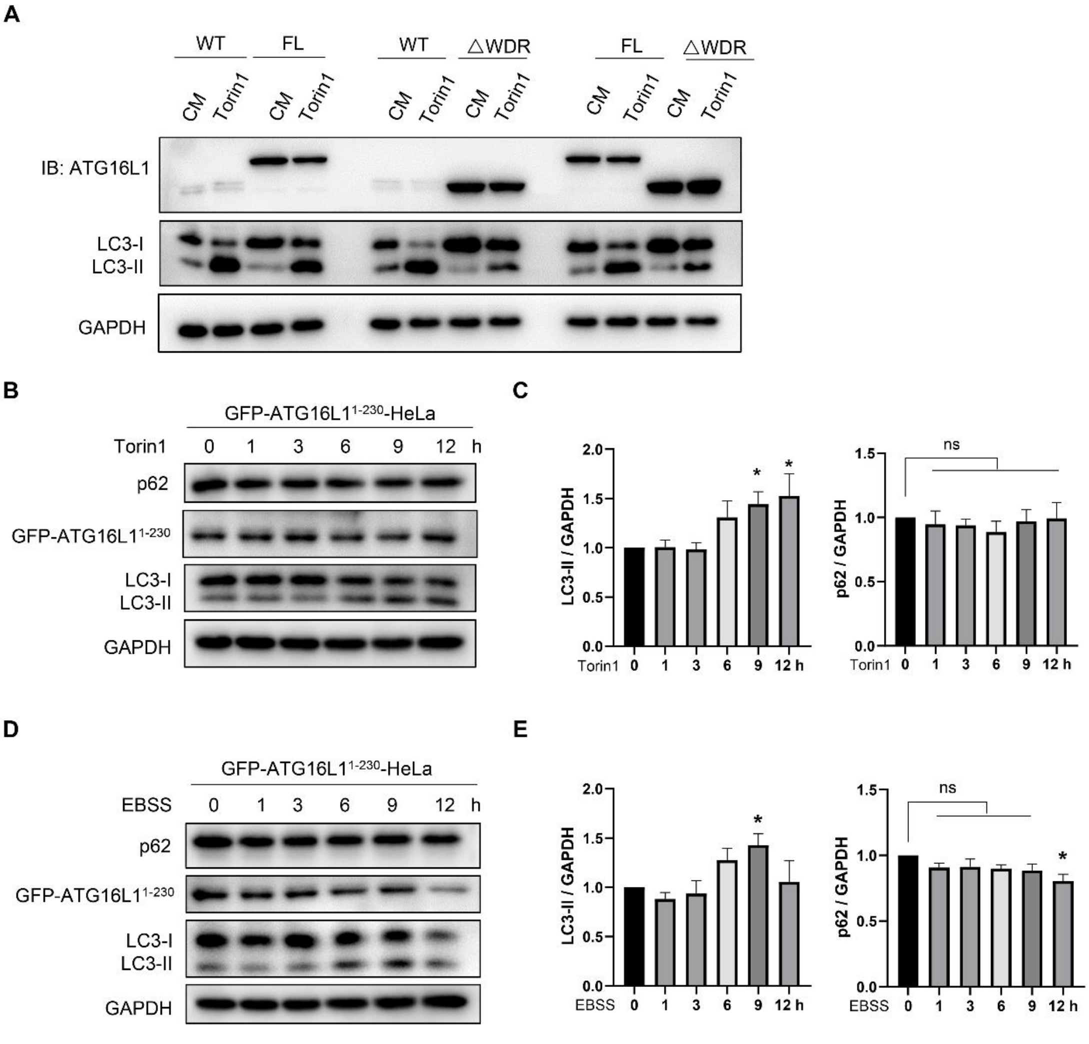

Figure S1. Canonical autophagy in ATG16L1$^{\Delta WDR}$ cells is blocked.

(**A**) WT-HeLa cells and *ATG16L1$^{-/-}$*-HeLa cells stably expressing GFP-ATG16L1 (FL) GFP-ATG16L1$^{\Delta WDR}$ (ΔWDR) were treated with 1 μM of torin1 for 12 h. ATG16L1, LC3, and GAPDH were analyzed by immunoblotting.

(**B-C**) ATG16L1$^{-/-}$-HeLa cells stably expressing GFP-ATG16L1$^{1\text{-}230}$ were treated with 1 μM of torin1 for 0, 1, 3, 6, 9, 12 h (B). GFP-ATG16L1$^{1\text{-}230}$, p62, LC3, and GAPDH were analyzed by immunoblotting (C). n=3. *P<0. 05

(**D-E**) ATG16L1$^{-/-}$-HeLa cells stably expressing GFP-ATG16L1$^{1\text{-}230}$ were treated with EBSS for 0, 1, 3, 6, 9, 12 h (D). GFP-ATG16L1$^{1\text{-}230}$, p62, LC3, and GAPDH were analyzed by immunoblotting (E). n=3. *P<0. 05

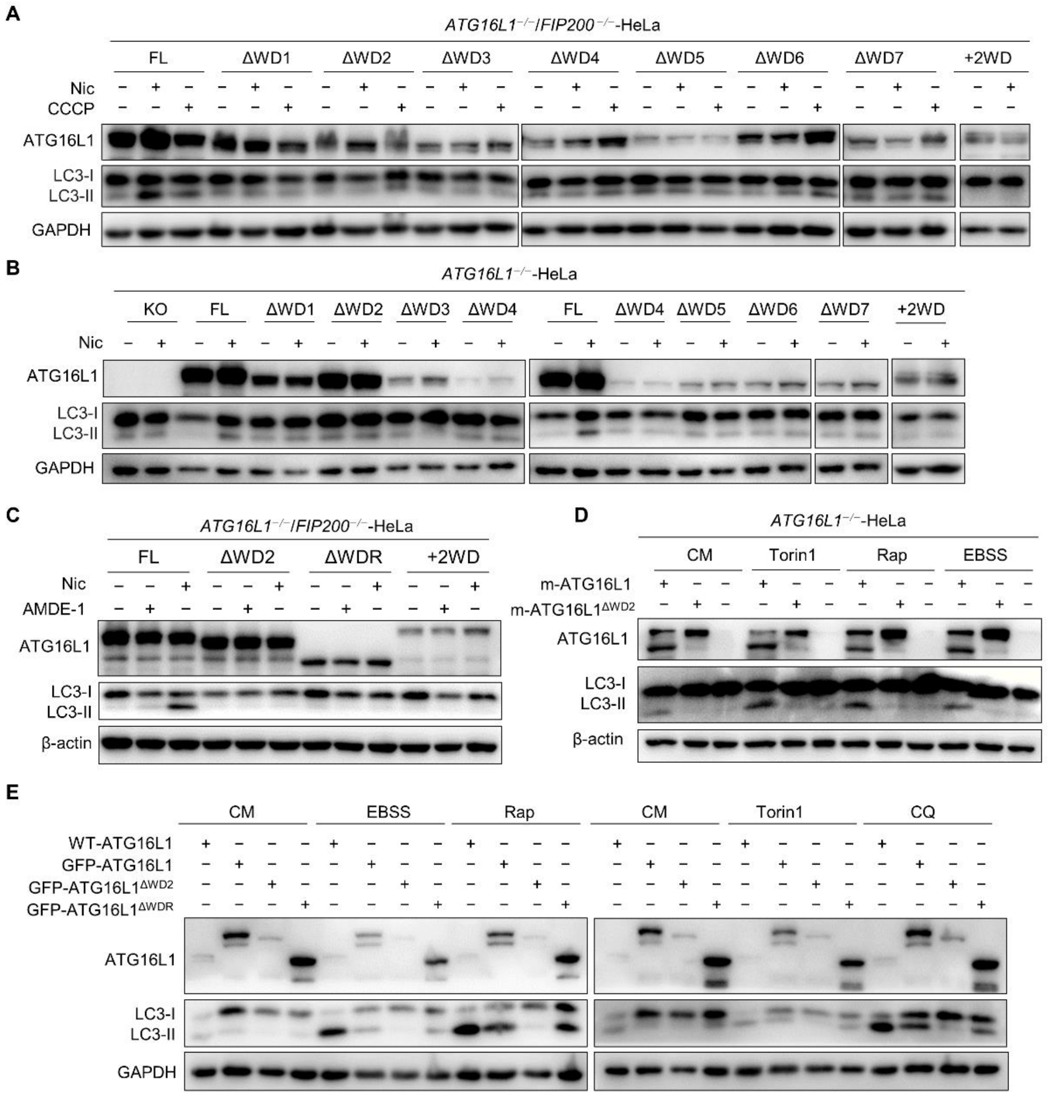

Figure S2. ATG16L1 with incomplete WDR could not mediate NCA induced by compounds.

(**A-B**) Immunoblot of ATG16L1, LC3, GAPDH in *ATG16L1*$^{-/-}$/*FIP200*$^{-/-}$-HeLa (A) and *ATG16L1*$^{-/-}$-HeLa (B) cells transiently transfected with ATG16L1 mutants for 42 h and then treated with 10 μM of niclosamide (Nic) or 30 μM of CCCP for 6 h.

(**C**) Immunoblot of ATG16L1 and LC3 in *ATG16L1*$^{-/-}$/*FIP200*$^{-/-}$-HeLa cells transiently transfected with ATG16L1 mutants for 42 h and then cells were treated with 10 μM of niclosamide (Nic) and AMDE-1 for 6 h.

(**D**) *ATG16L1*$^{-/-}$-HeLa cells were transiently transfected with mcherry-ATG16L1 or mcherry-ATG16L1$^{\Delta WDR}$ for 46 h and then treated with 1 μM of torin1, 1 μM of Rap (rapamycin), EBSS for 6 h. ATG16L1 and LC3 were analyzed by immunoblot.

(**E**) WT-HeLa cells and *ATG16L1*$^{-/-}$-HeLa cells stably expressing GFP-ATG16L1,

GFP-ATG16L1$^{\Delta WD2}$, and GFP-ATG16L1$^{\Delta WDR}$ were treated with EBSS, 1 μM of rapamycin (Rap), 1 μM of torin1, or 40 μM of chloroquine (CQ) for 6 h. ATG16L1 and LC3 were analyzed by immunoblot.

A

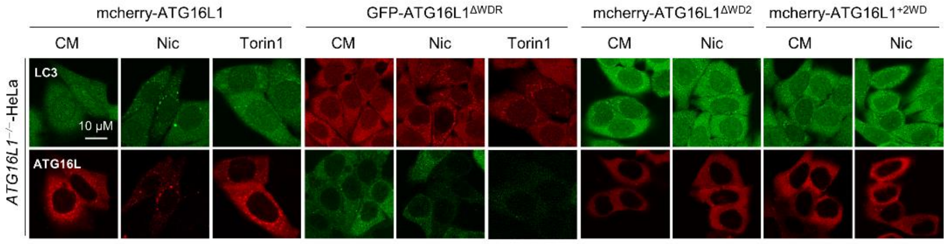

Figure S3. ATG16L1 with incomplete WDR could not mediate the formation of LC3 puncta in canonical autophagy.

(**A**) Confocal images of mcherry-ATG16L1 mutants and LC3 in *ATG16L1*$^{-/-}$-HeLa cells transiently transfected ATG16L1 mutants for 42 h and then treated with 10 μM of Nic (niclosamide) or 1 μM of torin1 for 6 h. Cells were stained for LC3, scale bar=10 μm.

Table S1. Vectors and primers information of ATG16L1 plasmid.

Gene	vector	forward primer (a) (5'-3')	primer b (5'-3')	primer c (5'-3')	reverse primer (d) (5'-3')
ATG16L1	plvx-acGFP-N1	ATGTCGTCGGGCCTCCGCGCCGCTGAC	/	/	GTACTGTGCCCACAGCACAGC
ATG16L1$^{\Delta WDR}$	plvx-acGFP-N1	ATGTCGTCGGGCCTCCGCGCCGCTGAC	/	/	ATCGAAGACACACAAGGCAGTAGC
ATG16L1	pmCherry-C1	ATGTCGTCGGGCCTCCGCGCCGCTGACTTC	/	/	TCAGTACTGTGCCCACAGCACAGC
ATG16L1$^{\Delta WD1}$	pmCherry-C1	ATGTCGTCGGGCCTCCGCGCCGCTGACTTC	TAGGGAACCGACACACAAGGCAGTAGC	TTGTGTGTCGGTTCCCT ATCTGGCAG	TCAGTACTGTGCCCACAGCACAGC
ATG16L1$^{\Delta WD2}$	pmCherry-C1	ATGTCGTCGGGCCTCCGCGCCGCTGACTTC	TCCCGTGAGCTTGAACTCACATTTTTCTC	GAGTTCA AGCTCACG GGACACAGTG	TCAGTACTGTGCCCACAGCACAGC
ATG16L1$^{\Delta WD3}$	pmCherry-C1	ATGTCGTCGGGCCTCCGCGCCGCTGACTTC	TGCAAACACTGTGTGCCGTAATCGATAATC	CGGCA CACAGTGTTTGCAGGATCCAG	TCAGTACTGTGCCCACAGCACAGC
ATG16L1$^{\Delta WD4}$	pmCherry-C1	ATGTCGTCGGGCCTCCGCGCCGCTGACTTC	CAGCTCCATTGTCTTTATGCAGACTTTGC	ATAAAGACAATG GAGCTGTTGGGAAAG	TCAGTACTGTGCCCACAGCACAGC
ATG16L1$^{\Delta WD5}$	pmCherry-C1	ATGTCGTCGGGCCTCCGCGCCGCTGACTTC	TGCACTGAACTCTCGAACTATGCTCTC	GT TCGAGAGTTCAGTGCACCTGGGTTC	TCAGTACTGTGCCCACAGCACAGC
ATG16L1$^{\Delta WD6}$	pmCherry-C1	ATGTCGTCGGGCCTCCGCGCCGCTGACTTC	GTGCTGCTTTGTCTGCTTGATAGC	CAAGCAGACAAAGCAG CACAGCTCATC	TCAGTACTGTGCCCACAGCACAGC
ATG16L1$^{\Delta WD7}$	pmCherry-C1	ATGTCGTCGGGCCTCCGCGCCGCTGACTTC	/	/	TCAGTACTGTGCCCACAGCACAGC
ATG16L1^{+2WD}	pmCherry-C1	ATGTCGTCGGGCCTCCGCGCCGCTGACTTC	CACTTTACTTTGTACTGTGCCCACAGCACAGC	GCACAGTACAAAGTAAAGTGTCCTG	TCAGAACATCTTGACGTTGTTC
